# The Robust Restriction of Zika Virus by Type-I Interferon in A549 Cells Varies by Viral Lineage and Is Not Determined by IFITM3

**DOI:** 10.3390/v12050503

**Published:** 2020-05-02

**Authors:** Theodore A. Gobillot, Daryl Humes, Amit Sharma, Caroline Kikawa, Julie Overbaugh

**Affiliations:** 1 Division of Human Biology, Fred Hutchinson Cancer Research Center, Seattle, WA 98109, USA; tgobillo@fredhutch.org (T.A.G.); dhumes@fredhutch.org (D.H.); sharma.157@osu.edu (A.S.); ckikawa@fredhutch.org (C.K.); 2 Medical Scientist Training Program, University of Washington School of Medicine, Seattle, WA 98195, USA

**Keywords:** Zika virus, IFITM3, type-I interferon, interferon-stimulated genes

## Abstract

Type-I interferon (IFN-I) is a major antiviral host response but its impact on Zika virus (ZIKV) replication is not well defined, particularly as it relates to different circulating strains. Interferon stimulated genes (ISGs) that inhibit ZIKV, such as IFITM3, have been identified largely using overexpression studies. Here, we tested whether diverse ZIKV strains differed in their susceptibility to IFN-I-mediated restriction and the contribution of IFITM3 to this restriction. We identified a robust IFN-I-mediated antiviral effect on ZIKV replication (>100-fold reduction) in A549 cells, a commonly used cell line to study ZIKV replication. The extent of inhibition depended on the IFN-I type and the virus strain tested. Viruses from the American pathogenic outbreak were more sensitive to IFNα (*p* = 0.049) and IFNβ (*p* = 0.09) than African-lineage strains, which have not been linked to severe pathogenesis. Knocking out IFITM3 expression did not dampen the IFN-I antiviral effect and only high overexpression of IFITM3 led to ZIKV inhibition. Moreover, IFITM3 expression levels in different cells were not associated with IFN-mediated ZIKV inhibition. Taken together, our findings indicate that there is a robust IFN-I-mediated antiviral effect on ZIKV infection, particularly for American viruses, that is not due to IFITM3. A549 cells, which are a commonly used cell line to study ZIKV replication, present an opportunity for the discovery of novel antiviral ISGs against ZIKV.

## 1. Introduction

The recent spread and severe pathogenic features of Zika virus (ZIKV) in the Americas have highlighted the epidemic potential of this emerging pathogen. The American outbreak clade of ZIKV strains has been linked to fetal abnormalities, a severe congenital syndrome in neonates, and adverse neurological outcomes in adults [1,2,3,4]. Prior to the American epidemic, documented outbreaks of ZIKV were rare [5,6,7].

ZIKV was first identified in Africa over 70 years ago. There is limited evidence that African-lineage ZIKV infections are associated with the severe pathogenic profile that has been fueled by the American clade, which clusters with Asian-lineage strains. This raises the possibility that African- and Asian-lineage ZIKV strains may have distinct pathogenic properties.

Type-I interferon (IFN-I) is a critical component of the host innate immune response to viral infection [8]. Upon recognition of viral infection, cells enter a transcriptional program that increases the production of IFN-I (IFNα and IFNβ), which establishes an anti-viral state in bystander cells and restricts viral replication in infected target cells [9]. The ability of IFN-I to restrict viral replication is largely due to the activation of hundreds of interferon-stimulated genes (ISGs) that have a wide range of anti-viral functions [10,11]. IFN-I is capable of restricting ZIKV in cell culture [12,13], and most murine models of ZIKV infection and pathogenesis require ablation of the IFN-I signaling pathway, underscoring the important role of ISGs in restricting ZIKV replication [14]. One such ISG is the Interferon-Induced Transmembrane Protein 3 (IFITM3), which was the first ISG described as a key effector of the IFN-I response against ZIKV in a variety of cells including A549, HeLa, 293T, HDFa, and HFF cells [15,16,17]. IFITM3 is a small transmembrane protein that restricts a broad array of viruses and is potently induced by IFN-I [18]. It is unclear whether strains of ZIKV differ in their susceptibility to IFN-I-mediated restriction and/or restriction by IFITM3.

The goals of this study were to determine whether ZIKV strains differ in their susceptibility to restriction by IFN-I and how IFITM3 contributes to the antiviral effects of IFN-I on ZIKV replication. Using a panel of nine ZIKV strains, we found that the African-lineage viruses were less sensitive to the effects of IFN-I than the Asian-lineage viruses. We also found that IFITM3 does not explain the IFN-I-mediated restriction of African or Asian-lineage viruses in A549 cells.

## 2. Materials and Methods

### 2.1. Viruses

ZIKV strains were kindly provided by BEI Resources (MR 766, IbH 30656, PRVABC59, FLR, H/PAN/2016/BEI-259634, H/PAN/2015/CDC-259359) and Michael Diamond (DAK-AR-25, DAK-AR-67, DAK-AR-71). All ZIKV strains were propagated in Vero cells at an MOI of 0.01, as previously described [19]. Viral titers were determined by the TCID50 assay described below. Experiments were performed with aliquots that had undergone at most two freeze-thaw cycles, which was not found to have any discernible effect on viral titers.

### 2.2. Cells

A549 cells (A. Berger; ATCC) were maintained in RPMI (Invitrogren) supplemented with 10% fetal bovine serum (FBS), 2 mM l-glutamine, and 1× Anti-anti (anti-microbial/anti-mycotic, Gibco). Vero cells (A. Geballe; ATCC), HEK293T cells, Jeg3 cells (ATCC), and SNB-19 cells (ATCC) were maintained in DMEM (Invitrogen) supplemented with 10% FBS, 2 mM l-glutamine, and 1× Anti-anti. SH-SY5Y cells (ATCC) were maintained in EMEM (Invitrogen) supplemented with 15% FBS, 2mM l-glutamine, and 1× anti-anti. The identity of the A549 cells and HEK293T cells was confirmed using STR CODIS fingerprinting and these cell lines were found to be mycoplasma-free by the Research Cell Bank shared resource at the Fred Hutchinson Cancer Research Center.

### 2.3. Sequencing and Phylogenetic Analysis of ZIKV Strains

All ZIKV stocks were sequence-confirmed by Sanger sequencing of a 1.8–3.4-kbp region of the ZIKV genome that encodes non-structural proteins 1 through 3. To do this, viral RNA was isolated using the QiaAMP Viral RNA Mini Kit (Qiagen) and cDNA was produced using SuperScript III First Strand Synthesis System (Invitrogen) with random hexamers according to the manufacturer’s suggested protocol. The Primal Scheme primer designer software (http://primal.zibraproject.org/) was then used to design primers that tiled across the complete open reading frame in ≈645 bp fragments that overlapped by ≈210 bp [20]. A subset of these primers was used to generate two overlapping sub-amplicons by PCR amplification of cDNA with Q5 ReadyMix (NEB) (Table S1). Thermocycling conditions used were as follows:

1. 98 °C, 30 s
2. 30 cycles:
  98 °C, 15 s
  65 °C, 5 min

The sub-amplicons were then subjected to Sanger sequencing using primers that bind within each sub-amplicon and sequences were confirmed for all strains with published sequences. (Table S2). Full-length open-reading-frame nucleotide sequences of ZIKV strains in the panel, as well as other ZIKV strains, were used to construct a maximum-likelihood phylogenetic tree with PhyML using a general time-reversible nucleotide substitution model [21].

### 2.4. IFN-I Sensitivity Assay

To measure the impact of IFN-I treatment on ZIKV replication, 8 × 10^4^ A549, Jeg3, SNB-19, or SH-SY5Y cells were either left untreated or pretreated with 1000 U/mL of IFNα-2a or IFNβ (PBL Assay Science, carrier-free) for 24 h in each well of a 24-well plate. After pretreatment, cells were infected at an MOI of 1 in a final volume of 250 µL of serum-free RPMI for 4–6 h. The inoculum was then aspirated and replenished with 1 mL of complete RPMI without IFN-I or containing 1000 U/mL of IFNα-2a or IFNβ. At 48 h post-infection (hpi), 250 uL of supernatants were harvested and cleared of cellular debris at 4 °C at 300G for 10 min and 2 × 100 uL aliquots were stored at −80 °C until titration by TCID50 assay. For the data analysis, all values were plotted, and statistical analyses performed using Prism version 7 (GraphPad Software, San Diego, CA, USA). Percent Relative Infection was determined by dividing the TCID50 titer in the IFNα- or IFNβ-treated sample by the untreated sample.

### 2.5. TCID50 Assays

ZIKV titers were determined by TCID50 assay on Vero cells in a 96-well format. One day prior to titration, Vero cells were seeded in 100 uL of complete DMEM in a flat-bottomed 96-well plate at 8 × 10^3^ cells per well. For each condition tested, seven serial 10-fold dilutions of viral supernatants were prepared, starting at a concentration of 1 uL/well, with each dilution including 10 replicate wells and two mock infected wells. Cells were infected with 50 uL of each viral dilution in serum free DMEM for 4–6 h, before being replenished with 100 uL of DMEM with 3% FBS, for a final concentration of 2% FBS. On day 5 post-infection the wells at a given dilution were scored by light microscope for the presence or absence of cytotoxicity and the TCID50/mL was calculated using the Spearman–Karber method. 

### 2.6. ZIKV E-Protein Staining

Monoclonal Anti-Flavivirus Group Antigen (4G2) antibody (ATCC) was conjugated with APC (Novus Lighting-Link) according to manufacturer’s protocol. For each condition, 3 × 10^5^ cells were plated in a single-well of a 6-well dish. Cells were subsequently infected with 1 mL of inoculum (MOI 1) for 4–6 h, before being replenished with 3 mL of RPMI-10% FBS-2 mM l-glutamine-1× Anti-anti (anti-microbial/anti-mycotic, Gibco). Cells were harvested at 24 h post-infection and were subsequently fixed and permeabilized with the BD Fixation/Permeabilization solution kit according to the manufacturer’s protocol (BD Biosciences). Intracellular E-protein staining was then carried out with 0.25 µg of APC-conjugated 4G2 antibody per condition. Cells were washed twice in Perm/Wash buffer and resuspended in PBS prior to flow cytometry analysis on a BD FACSCanto II flow cytometer. All data was analyzed using FlowJo v10 software (BD, Franklin Lakes, NJ, USA).

### 2.7. Generation of Stable Cell Lines Overexpressing IFITM3

IFITM3-expressing A549 cells were generated as previously described [22]. Briefly, the N-terminal FLAG-tagged IFITM3 open-reading frame was cloned into pHIV-Zsgreen [23] (Addgene plasmid # 18121, a gift from Bryan Welm and Zena Werb) directly upstream of the IRES-driven ZsGreen fluorescent reporter, thus linking IFITM3 transcript expression to ZsGreen expression. Virus-like particles (VLPs) were generated in HEK293T cells by co-transfecting cells with pHIV-ZsGreen constructs (either IFITM3-encoding or empty vector as control), psPAX2 (Addgene plasmid # 12260, an HIV-based packaging plasmid gifted from Didier Trono), and pMD2.G (Addgene plasmid # 12259, vesicular stomatitis virus glycoprotein (VSV-G) envelope plasmid gifted from Didier Trono) at a ratio of 1:1:0.5 using FuGENE 6 (Promega) according to the manufacturer’s protocol. Supernatants from HEK293T cells were collected 48 h post-transfection and concentrated ≈100-fold using Amicon Ultracel 100 K filters (Millipore). VLPs were then used to transduce A549 cells that has been plated 24 h prior in a 6-well plate at 1 × 10^5^ cells/well in 2 mL of RPMI supplemented with 10% FBS and 2 mM glutamine. A549 cells were transduced by spinoculation at 1200× *g* for 90 min. The following day, the cells were expanded into new T75 flasks and were subsequently passaged and maintained in complete DMEM. IFITM3-expressing cells were sorted by gating cells in the 50th-percentile of zsGreen expression on a FACSAria II cell sorter.

### 2.8. Generation of Clonal Cell Lines Expressing Different Levels of IFITM3

Monoclonal cell populations of IFITM3-expressing A549 cells (generated as described above) were isolated by limiting dilution. Briefly, IFITM3-expressing A549 cells were seeded at a density of one cell per well in a 96-well plate in 150 µL of RPMI-10% FBS-2mM l-glutamine-1× Anti-anti (anti-microbial/anti-mycotic, Gibco). Seven days after plating, single colonies could be visualized, and the media was changed on all wells. Ten days after plating, the number of colonies in each well were tallied and wells that contained only a single colony were selected for further analysis. Cells from wells containing single colonies were trypsinized when they were close to confluency (≈15 days after plating) and expanded into a well of a 24-well plate. Clonal cell populations were subsequently screened for zsGreen mean fluorescence intensity and two cell lines (IFITM3-rel and IFITM3-high) were selected to use in experiments.

### 2.9. Generation of IFITM3 and IRF9 Knockout Cell Lines and Validation by TIDE Analysis

For generation of IFITM3-knockout and IRF9-knockout A549 cell lines, guide RNAs targeting the first exon of Ifitm3 and the third exon of Irf9, or non-targeting control guide RNA, were cloned into pLentiCRISPR (Addgene plasmid # 49535, a gift from Feng Zhang) [24]. VLPs were generated by co-transfecting HEK 293Ts with the pLentiCRISPR plasmids, the psPAX2 packaging vector, and pMD2.G and harvested and concentrated as described above. A549 cells were transduced with pLentiCRISPR VLPs encoding and maintained as described above, except that cells were treated with 2 μg/mL puromycin to select for sgRNA and Cas9 expression 2 days after being moved to T75 flasks. The two IFITM3-targeting sgRNAs that yielded the most efficient knockout of IFITM3 were sgRNA1, 5′-GCAGCAGGGGTTCATGAAGA-3′; and sgRNA2, 5′-TTGAGCATCTCATAGTTGGG-3′. The IRF9-targeting sgRNA was 5′-ACAATTCCACAGGCCAGCCA-3′ and the non-targeting control was 5′-ATCTCGGGTCGACTGCGGAT-3′. Gene knockout was characterized by TIDE analysis. Briefly, after three rounds of puromycin selection, genomic DNA was isolated. For IFITM3-knockout cell lines, DNA was isolated using QuickExtract DNA extraction solution (Lucigen) by resuspending cells in 100 μL of the solution, and by denaturing for 20 min at 60 °C and 20 min at 95 °C. The ifitm3 locus was amplified using the following primer set: forward 5′-ACCATCCCAGTAACCCGACCG-3′ and reverse 5′-GCTGATACAGGACTCGGCTCC-3′. For IRF9-knockout cell lines, DNA was isolated using a Qiagen Blood Mini kit per the manufacturer’s protocol. The Irf9 locus was amplified using the following primer set: forward 5′-CCTGCATAATCCCTTCTGAGC-3′ and reverse 5′-CCCTGGAGTTTCTGCTTCCT-3′. Amplicons were Sanger sequenced and gene editing was measured using TIDE analysis (https://tide-calculator.nki.nl/).

### 2.10. Western Blots and Quantification

Whole cell extracts were prepared by lysing the cells in RIPA cell lysis buffer (50 mM Tris pH 8.0, 0.1% SDS, 1% Triton-X, 150 mM NaCl, 1% deoxycholic acid, 2 mM PMSF). Standard Western blotting procedures were used with the following antibodies: IFITM3 (Proteintech 11714-1-AP, used at 1:1000 dilution), IFITM2 (Proteintech 66137-1-Ig, used at 1:500 dilution), FLAG (OriGene TA100023, used at 1:2000 to 1:5000 dilution), ISG15 (Cell Signaling 2743, used at 1:1000 dilution), and GAPDH (BioRad MCA4739P, used at 1:5000 dilution). Protein expression was quantified by measuring the band intensities using Image J.

### 2.11. Influenza A virus (IAV) and Murine Leukemia Virus (MLV) Virus-Like Particle (VLP) Infections

Influenza A virus (IAV) (generously provided by A. Russell and J. Bloom) is an mCherry-expressing reporter virus where HA is replaced with mCherry. For murine leukemia virus (MLV), reporter VLPs were made by packaging the lentiGuide.mCherry vector [25] (a gift from Richard Young, AddGene plasmid #104375) with psPAX2 and pseudotyping with an amphotropic MLV envelope. For both viruses, 8 × 10^4^ IFITM3-expressing and control cells were plated in a 24-well plate 1 day prior to infections in a final volume of 1 mL of complete RPMI. For IAV, cells were infected at an MOI of 10 in 500 µL of complete RPMI for 16 h. Cells were harvested and fixed in 1% paraformaldehyde. For MLV, cells were infected with a dilution of VLPs in complete RPMI supplemented with 10 µg/mL DEAE dextran. Cells were harvested and fixed in 1% paraformaldehyde 72 h post infection. Both IAV and MLV-infected cells were assessed for mCherry expression using a Fortessa X50 flow cytometer and data was analyzed using FlowJo v9 software.

### 2.12. Data Availability

The accession numbers for the ZIKV strains utilized in this study are KU963573 (MR 766), KU963574 (IbH30656), KU955591 (DAK-AR-25), MF510857 (DAK-AR-67), KU955595 (DAK-AR-71), KX198135 (H/PAN/2016/BEI-259634), KX156774 (H/PAN/2015/CDC-259359), KX087102 (FLR), KX087101 (PRVABC59).

## 3. Results

### 3.1. Effect of IFN-I Treatment on Diverse ZIKV Strains in A549 Cells

**Figure 1:**
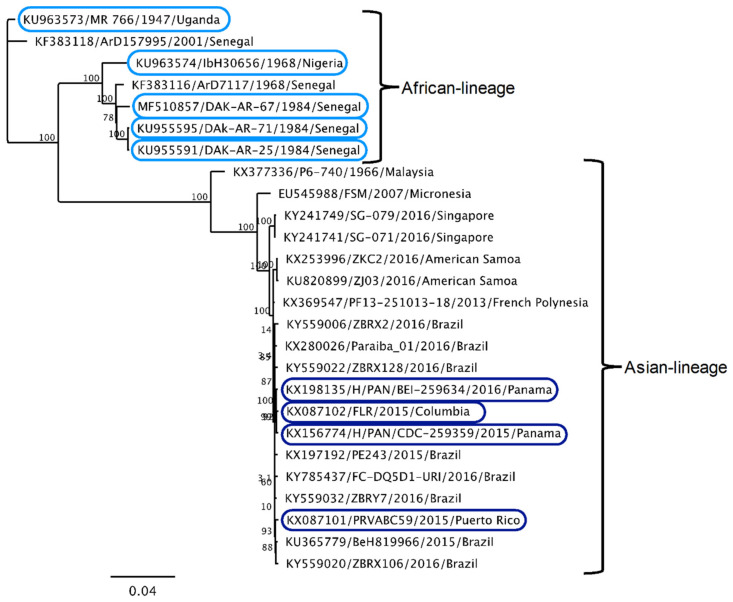
Phylogenetic relationships of Zika virus strains used in this study. Maximum-likelihood phylogeny of full-length open-reading-frame nucleotide sequences using Zika virus strains in this study (circles) and reference sequences isolated from humans, non-human primates, and mosquitoes. At least one representative strain from each documented Zika virus (ZIKV) clade is included in the phylogenetic tree.

In order to test the hypothesis that IFN-I sensitivity differs between African-lineage and Asian-lineage ZIKV strains, a panel of nine viruses was tested for their ability to replicate in A549 cells in the presence or absence of IFN-I. Five strains belong to the African lineage and four strains belong to the American outbreak clade within the Asian lineage (Figure 1, circles). The percent identity of the complete genomes of African vs. Asian lineage strains in this panel is 88%–89%, which is representative of the overall diversity of isolated ZIKV strains [6]. All Asian-lineage viruses were isolated from infected humans, while only one African-linage virus was isolated from an infected human (IbH 30656). Three African-lineage strains were isolated from mosquitoes (DAK-AR-25, DAK-AR-67, DAK-AR-71) and one from a sentinel rhesus macaque (MR 766) (Table 1). In addition, these strains have diverse passage histories. Most have undergone 3–5 passages in mosquito (AP61, C6/36) and/or African-green monkey (Vero) cell lines; however, MR 766 has been extensively passaged in mouse brain and subsequently in Vero cells. IbH 30656 has a similar but less extensive high-passage profile. The number of passages in AP61 cells for DAK-AR-67 and DAK-AR-71 is unknown.

**Figure 2:**
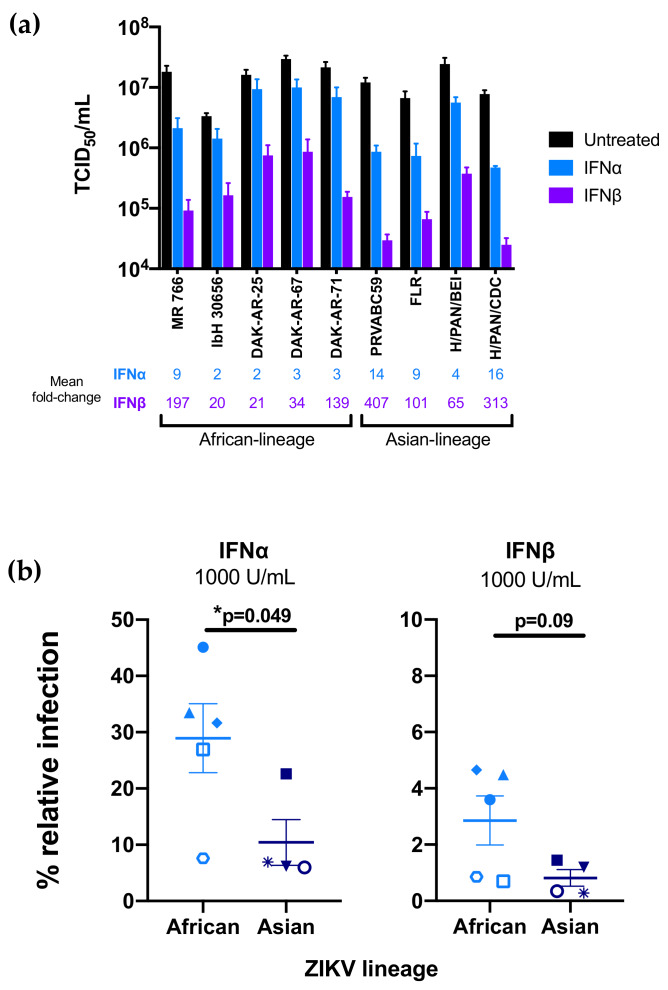
Effect of type-I interferon (IFN-I) pretreatment on diverse Zika virus strains in A549 cells. (**a**) The susceptibility of each ZIKV strain to restriction by IFNα-2a or IFNβ was assessed in A549 cells. Cells were treated with 1000 U/mL IFNα-2a or IFNβ 24 h prior to infection as well as following infection with each ZIKV strain. The titer (TCID50/mL) of each strain 48 h post-infection in the absence of IFN-I (black), presence of IFNα-2a (blue), and presence of IFNβ (purple) is shown. All data represent the average of at least four independent experiments that were carried out with two independently generated stocks of each ZIKV strain. Error bars represent SEM. The mean fold-reduction in viral replication of each ZIKV strain by pretreatment with IFNα and IFNβ is listed below the graph. (**b**) Comparison of IFNα-2a-mediated (1000 U/mL) and IFNβ-mediated restriction (1000 U/mL) of African-lineage vs. Asian-lineage ZIKV strains. Percent relative infection (IFN+/IFN-) is plotted for African-lineage (light blue) and Asian-lineage (dark blue) ZIKV strains. Error bars indicate SEM. A two-tailed Student’s *t*-test was used to compare percent relative infection of African-lineage vs. Asian-lineage ZIKV strains for each IFN condition (* *p* = 0.049).

For each strain in the panel, two independent stocks were amplified on Vero cells to account for any stock to stock variation, and the sensitivity of the viral stocks to pretreatment with IFNα-2a or IFNβ (1000 U/mL) in A549 cells was determined (Figure 2a). Both African-lineage and Asian-lineage viruses were more potently inhibited by IFNβ pretreatment than IFNα, with viral replication reduced 2–16-fold in response to IFNα and 20–407-fold in response to IFNβ (Figure 2a). The biggest differences were between IbH 30656/DAK-AR-25 (both African-lineage) and H/PAN/CDC-259359 (Asian-lineage) (≈8-fold) for IFNα and IbH 30656 and PRVABC59 (Asian-lineage, ≈20-fold) for IFNβ. There was a range of responses within each lineage: for example, among African-lineage strains, MR 766 isolate was most susceptible to both IFNα and IFNβ. Among Asian-lineage strains, H/PAN/CDC-259359 was the most sensitive to IFNα, while PRVABC59 was the most sensitive to IFNβ.

As an aggregate, African-lineage ZIKV strains were significantly less susceptible to IFNα restriction than Asian-lineage strains (Figure 2b; *p* = 0.049); they were also less susceptible to IFNβ, though differences in sensitivity to IFNβ (Figure 2b; *p* = 0.09) were only a statistical trend.

Taken together, the data reinforces IFN-I as a potent restrictor of ZIKV replication, albeit with substantial strain-to-strain differences in susceptibility, with African-lineage strains less sensitive to IFN-I than Asian-lineage strains.

### 3.2. Expression of IFITM3 at Levels Similar to IFN-Induction in A549 Cells Does not Restrict ZIKV

**Figure 3:**
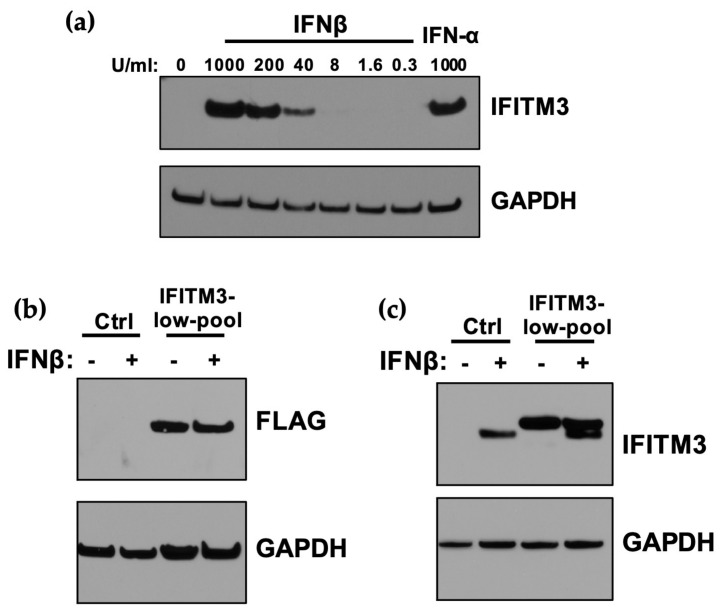
Expression of IFITM3 in A549 cells transduced with exogenous IFITM3 compared to after IFN-I-induction. (**a**) Western blot analysis of IFITM3 expression in A549 cells pretreated with increasing concentrations of IFNβ and 1000 U/mL IFNα for 24 h. The concentration of IFNβ is indicated above each lane. (**b**) Western blot analysis of IFITM3-FLAG expression using an anti-FLAG antibody in IFITM3-low-pool A549 cell lines. Control cells were either untreated or treated with IFNβ (1000 U/mL) for 24 h. (**c**) Western blot analyses of expression of IFITM3-FLAG protein compared to endogenous IFITM3 using an anti-IFITM3 antibody. Control cells were either untreated or treated with IFNβ (1000 U/mL) for 24 h.

Given the potent IFN-I-induced restriction of ZIKV strains in the panel, with a several hundred-fold reduction in replication for the most potently inhibited strains, we examined the contribution of IFITM3 to this IFN-I-mediated inhibitory effect, because it was the first reported antiviral ISG against ZIKV. IFITM3 was induced by both IFNβ and IFNα in A549 cells, with slightly higher levels (≈2-fold) in IFNβ than IFNα-treated cells at the same dose (1000 U/mL; Figure 3a). ISG15 was also induced by IFN-I treatment, with equivalent levels of induction when comparing A549 cells treated with 1000 U/mL of IFNα and IFNβ (Figure S1). The induction of IFITM3 was dose-dependent, as shown with increasing doses of IFNβ (Figure 3a). To determine whether the induction of IFITM3 expression could explain the sensitivity of ZIKV to IFN-I, an A549 cell line expressing an N-terminally FLAG-tagged IFITM3 was generated (Figure 3b). To ensure that the levels of IFITM3 were physiologically relevant, we sorted cells and selected cells with relatively lower levels of IFITM3 (IFITM3-low-pool cell line); these sorted A549 cells expressed similar levels of IFITM3 as IFNβ-treated A549 control cells (Figure 3c).

**Figure 4:**
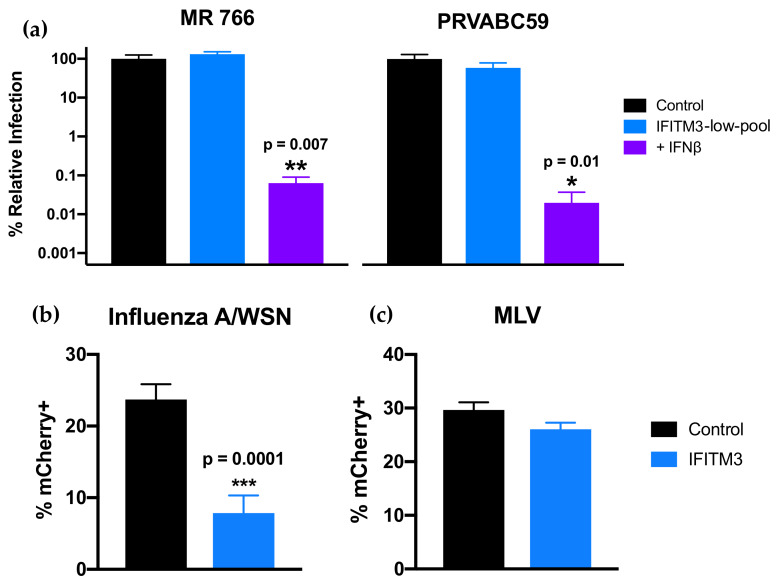
Infection of cells expressing IFITM3-FLAG in the absence and presence of IFNβ. (**a**) Infection of IFITM3-expressing cells with ZIKV strains MR 766 and PRVABC59. Viral titers 48 hpi in untreated (blue) or IFNβ pretreated (1000 U/mL; 24 h; purple) IFITM3 cells are shown. For each strain, percent relative infection (IFITM3-low-pool/Control or IFN+/Control) is shown. Data represent the average of four independent experiments that were carried out with two independently generated stocks of each ZIKV strain. Error bars indicate SEM. * *p* = 0.01, ** *p* = 0.007 (one-way analysis of variance (ANOVA) followed by Dunnett’s post-hoc test for multiple comparisons). (**b**,**c**) Infection of IFITM3-expressing cells with mCherry-expressing (**b**) influenza A virus (IAV) and (**c**) virus-like particles (VLPs) expressing murine leukemia virus (MLV) envelope protein. Data represent the average of at least four independent experiments. Error bars indicate SEM. *** *p* = 0.0001 (two-tailed Student’s *t*-test).

To assess the effect of IFITM3 expression on ZIKV replication, IFITM3-expressing and control cells were infected with African-lineage isolate MR 766 and Asian-lineage isolate PRVABC59, both of which were found to be especially susceptible to IFN-I (Figure 4). Viral replication was not significantly different in cells expressing IFITM3 than from control cells for either strain (Figure 4a). Importantly, IAV was significantly restricted in IFITM3-expressing cells (*p* < 0.0001), while VLPs expressing the MLV envelope protein were not significantly restricted in these cells (Figure 4b,c). This is consistent with published data showing IFITM3 restricts IAV but not MLV [26]. Notably, ZIKV was potently inhibited in the same cells engineered to express IFITM3 when they were treated with IFNβ, with drastic reductions in viral replication for both strains (1.6 × 10^3^–5.1 × 10^3^-fold) when compared to infection of untreated control cells (Figure 4a; *p* = 0.007 for MR 766, *p* = 0.01 for PRVABC59). This shows that ISGs induced by IFN-I other than IFITM3 were driving the potent antiviral response to ZIKV in these cells.

### 3.3. Overexpression of IFITM3 above IFN-I-Induced Levels in A549 Cells Restricts ZIKV

**Figure 5:**
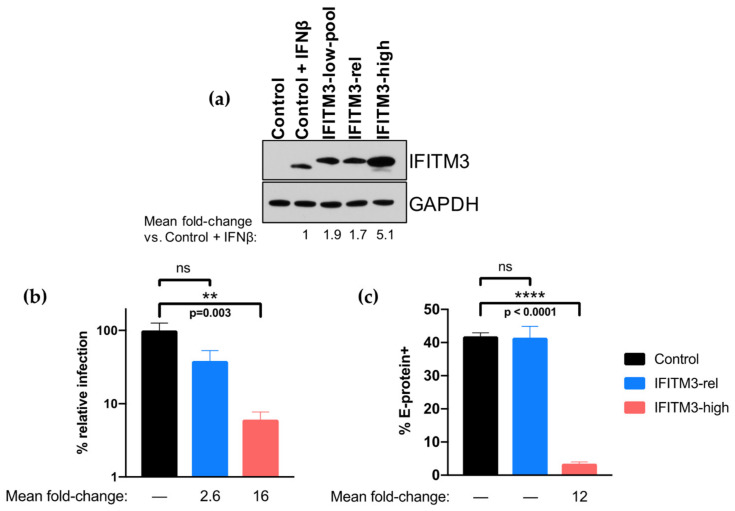
Infection of clonal A549 cells expressing IFN-relevant levels of IFITM3-FLAG or overexpressing IFITM3-FLAG. (**a**) Western blot analysis of IFITM3 expression using an anti-IFITM3 antibody in A549 control and clonal cell lines. Control cells were either untreated or treated with 1000 U/mL IFNβ for 24 h. IFITM3-rel cells are named for their IFN-I-relevant levels of IFITM3 expression, while IFITM3-high cells are named for their overexpression of IFITM3 above levels observed by IFN-I induction. The fold-change in IFITM3 expression compared to IFNβ-treated controls cells (normalized to GAPDH expression) is noted below the Western blot which was quantified by measuring the band intensities across three different exposures of the same Western blot and calculating the mean. (**b**,**c**) Infection of control (black), IFITM-rel (blue), and IFITM3-high (red) cell lines with ZIKV strain MR 766. The percent relative TCID50 titer of MR 766 measured at 48 hpi (**b**) and percent of E-protein-positive cells at 24 hpi (**c**) is shown. All data represent the average of at least four independent experiments. The mean fold-reduction in viral replication (**b**) or percent E-protein-positive cells (**c**) in each cell line as compared to control cells (Control/IFITM3-rel or Control/IFITM3-high) is presented below each graph. Error bars represent SEM. ** *p* = 0.003, **** *p* < 0.0001 (one-way analysis of variance (ANOVA) followed by Tukey’s post-hoc test for multiple comparisons).

Our results indicating that IFITM3 did not restrict ZIKV replication in our system contrasted with results of several studies that have described IFITM3 as a ZIKV restriction factor. One study that also used A549 cells to examine IFITM3-mediated restriction of ZIKV utilized systems that overexpressed IFITM3 at levels that were higher than seen upon IFN-I induction [16], as did a second study in 293T, HFF, and HDFa cell types [15]. To determine if these differences in expression levels could explain the differences in results, clonal A549 cell lines were generated that either overexpress IFITM3 above IFN-I-induced levels (5.1-fold higher; IFITM3-high cell line, Figure 5a) or express IFITM3 to levels that were closer and thus more relevant to those induced by treatment with IFN-I (1.7-fold higher; IFITM3-rel cell line, “-rel” for IFN-I-relevant, Figure 5a). These cells were infected with MR 766 in parallel to empty vector-transduced A549 control cells. While viral replication was not significantly different between control and IFITM3-rel cells, there was a significant reduction of viral replication in IFITM3-high cells (16-fold, *p* = 0.003; Figure 5b).

As the prior studies that suggested IFITM3 inhibits early stages of ZIKV replication used different time points and assays to study IFITM3-mediated restriction of ZIKV [15,16], we next determined whether we missed the activity of IFITM3 at earlier times or because of our methods of measuring virus. For this, we examined the percent of infected cells in control, IFITM3-rel, and IFITM3-high cells by E-protein staining at 24 h post-infection (Figure 5c). There was no difference in percent E-protein-positive cells between control and the low-expressing IFITM3-rel cells, while there was a 12-fold reduction in percent E-protein-positive cells in high-expressing IFITM3-high cells (*p* < 0.0001).

**Figure 6:**
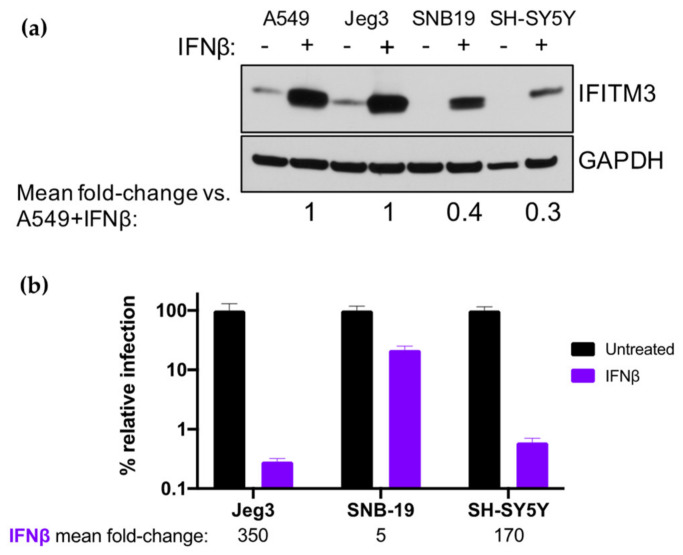
Effect of IFN-I pretreatment on ZIKV replication and expression of IFITM3 in Jeg3 and SNB-19 cells. (**a**) Western blot analysis of IFITM3 expression using an anti-IFITM3 antibody in A549, Jeg3, SNB-19, and SH-SY5Y cells. Cells were either untreated or treated with 1000 U/mL IFNα or IFNβ for 24 h. For each cell line, the relative induction of IFITM3 expression by IFNβ (normalized to GAPDH expression) as compared to A549 cells pretreated with IFNβ is noted below the Western blot. (**b**) Infection of Jeg3, SNB-19, and SH-SY5Y cells with ZIKV strain MR 766. The percent relative TCID50 titer measured at 48 hpi is shown. The mean fold-reduction in viral replication in each cell line by pretreatment with IFNβ is listed below the graph. Data represent the average of four independent experiments.

To rule out that the reason physiological IFN-I-induced levels of IFITM3 are not associated with restriction of ZIKV in A549 cells is because they are unusually low, we compared their IFN-I-induced IFITM3 levels to other cells, including glial (SNB-19), neuronal (SH-SY5Y), and placental cells (Jeg3) which play important roles in the neurological pathogenesis and vertical transmission of ZIKV [27,28,29,30]. IFN-I-induced levels of IFITM3 in Jeg3 cells were similar to those observed in A549 cells, while SNB-19 and SH-SY5Y cells had lower levels of IFN-I-induced IFITM3 induction (0.3–0.4-fold; Figure 6a). ZIKV was potently restricted by IFN-I treatment in Jeg3 and SH-SY5Y cells (350-fold and 170-fold reduction in viral titers, respectively), but less potently in SNB-19 cells (5-fold reduction; Figure 6b). Overall, the levels of IFITM3 were not associated with IFN-I-mediated inhibition of ZIKV across these cell lines. This is exemplified by the lower levels of IFITM3 in SH-SY5Y cells vs. A549 cells, yet similar levels of IFN-I-mediated inhibition. These findings indicate that low levels of IFITM3 induction do not explain the lack of restriction in A549 cells. Collectively, these results demonstrate an inhibitory effect of IFITM3 at very high levels that does not reflect what happens at physiological IFN-I-induced levels in these cell types.

### 3.4. CRISPR-Cas9 Inactivation of IFITM3 Does not Ablate the Effect of IFN-I on ZIKV Replication in A549 Cells

**Figure 7:**
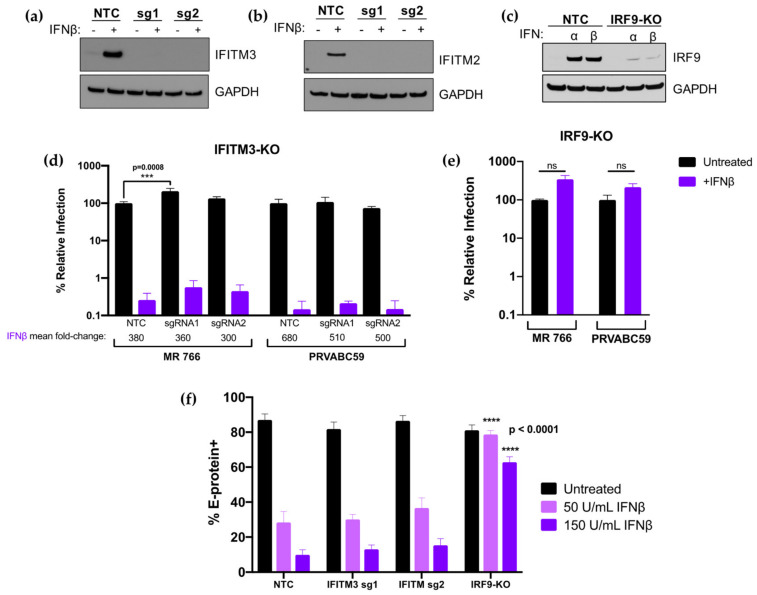
Analysis and infection results of IFITM3 and IRF9 knock-out cells. (**a**,**b**) Western blot analysis of IFITM3 (**a**) and IFITM2 (**b**) expression in untreated or IFNβ pretreated (1000 U/mL; 24 h) A549 cells transduced with non-targeting control (NTC), sgRNA1 (sg1), or sgRNA2 (sg2). The sgRNA used to transduce each cell line is indicated above each lane. (**c**) Western blot analysis of IRF9 expression in untreated, IFNα pretreated (1000 U/mL; 24 h), or IFNβ pretreated (1000 U/mL; 24 h) A549 cells transduced with NTC or an IRF9-targeting sgRNA. (**d**,**e**) Infection results with MR 766 and PRVABC59 showing viral titers 48 hpi in IFITM3-knockout (**d**) and IRF9-knockout (**e**) A549 cells. The percent relative infection (normalized to untreated NTC) of each strain in the absence of IFN-I (black) and presence of 1000 U/mL of IFNβ (purple; 24 h pretreatment) is shown in each indicated cell type. All data represent the average of four independent experiments. Error bars indicate SEM. *** *p* = 0.0008 (two-way analysis of variance (ANOVA) followed by Dunnet’s post-hoc test for multiple comparisons); ns = not significant (two-tailed Student’s *t*-test for each strain). (**f**) Infection results with MR 766 showing percent E-protein-positive cells 24 hpi in IFITM3-knockout, IRF9-knockout, and NTC A549 cells. The percent E-protein-positive cells in the absence of IFN-I (black) and presence of 50 U/mL IFNβ (light purple; 24 h pretreatment) and 150 U/mL IFNβ (dark purple; 24 h pretreatment) is shown in each indicated cell type. All data represent the average of four independent experiments. Error bars indicate SEM. All data comparisons were made to identical conditions in the NTC. **** *p* < 0.0001 (one-way ANOVA followed by Dunnett’s post-hoc test for multiple comparisons).

To better define the contribution of IFITM3 to the overall IFN-I response against ZIKV replication, we employed a complementary CRISPR-Cas9 gene editing approach to knock out IFITM3. In parallel, we inactivated IRF9 because it is an essential component of the IFN-I signaling cascade and thus serves as a control for ablation of the IFN-I response. The bulk transduced cell lines had high percent total editing of the IFITM3 loci, including two different sgRNAs to IFITM3 (IFITM3 sgRNA1: 96%; IFITM3 sgRNA2: 88%; IRF9: 100%). Cells transduced with IFITM3-targeting sgRNA1 or sgRNA2 were depleted in IFITM3 expression as compared to cells transduced with a non-targeting control (NTC) sgRNA, both basally and when treated with IFNβ (Figure 7a). Due to the high level of sequence identity between IFITM2 and IFITM3, IFITM2 expression was also knocked out in cells transduced with IFITM3-targeted sgRNAs (Figure 7b). Thus, the IFITM3-knockout cell lines could detect the loss of activity of either IFITM3 or IFITM2 against ZIKV. Cells transduced with the IRF9-targeting sgRNA were depleted in IRF9 expression (Figure 7c).

IFITM3-knockout, IRF9-knockout, and NTC cells were infected with an African-lineage (MR 766) or Asian-lineage (PRVABC59) virus at an MOI of 1. In untreated cells, there was a modest but statistically significant (2.5-fold; *p* = 0.0008) increase in virus replication of MR 766 in sgRNA1 IFITM3-knockout cells that was not observed in sgRNA2 IFITM3-knockout cells or in either of the IFITM3-knockout cells infected with PRVABC59. In IFN-treated cells, there was no significant impact on the level of IFNβ inhibition with the ablation of IFITM3 expression. Viral titers were reduced by very similar amounts in control cells (380-fold) compared to IFITM3 knock-out cells (300- and 360-fold) for MR766 infection and in control cells (680-fold) and IFITM3 knock-out cells (510- and 500-fold) for PRVABC59, (Figure 7d, purple bars). The effect of IFNβ was completely abrogated in IRF9-knockout cells, as expected (Figure 7e).

We also examined whether the time of infection or assay read-out impacted the results in our IFITM3 knockout cells by examining E-protein staining at 24 h post-infection (Figure 7f). In these experiments, two lower doses (50, 150 U/mL) of IFNβ were used to enhance the dynamic range of the assay. In NTC cells, E-protein staining was 3.1- and 8.8-fold lower at 50 and 150 U/mL IFNβ, respectively. We observed a similar level of inhibition in IFITM3 knockout cells: 2.7- (sgRNA1) and 2.4-fold (sgRNA2) inhibition at 50 U/mL; 6.3- (sgRNA1) and 5.6-fold (sgRNA2) inhibition at 150 U/mL (Figure 7f). The differences between untreated or IFN-treated conditions between NTC and IFITM3-knockout cells were not significant. IFNβ-mediated restriction of ZIKV at both IFNβ concentrations was again abrogated in IRF9-knockout cells as compared to NTC cells (*p* < 0.0001 at both IFN concentrations).

Taken together, these data suggest at best a very modest contribution of IFITM3 to the overall IFN-I response to ZIKV in A549 cells, which is consistent with the results in cells exogenously expressing IFITM3. Thus, the several order of magnitude restriction of ZIKV replication, including both African and Asian lineage strains, in A549 cells is due to ISGs other than IFITM3. 

## 4. Discussion

The emergence of more prevalent and severe pathogenic outcomes associated with ZIKV infection has brought renewed interested in the study of this virus over the past few years. Given the importance of IFN-I in the antiviral response, we asked whether African- and Asian-lineage ZIKV strains differ in their IFN-I sensitivity and whether the previously defined ZIKV antiviral protein IFITM3 plays a critical role in the IFN-I response. ZIKV strains were potently inhibited by IFN-I in A549 cells, although they had a range of susceptibilities and African-lineage strains were less sensitive to IFN-I-mediated restriction than Asian-lineage strains. The potent IFN-I antiviral activity was not due to IFITM3 in A549 cells, suggesting that these cells could shed light on novel ISGs with the potential to restrict ZIKV replication, including those that determine lineage-dependent differences in the IFN-I antiviral effect.

African-lineage strains were significantly less sensitive to the effects of IFNα and showed a trend for a difference with IFNβ compared to Asian-lineage strains. This was at first counter-intuitive given the fact that it is Asian-lineage strains that cause severe neuropathologic outcomes in fetuses, neonates, and adults. However, the data are in line with several recent studies that have demonstrated that infection with African-lineage strains resulted in enhanced replication kinetics, virus production, cytopathicity, and disease progression in murine models [14,31,32,33,34,35,36,37,38,39,40,41,42,43]. Further, one such study showed that induction of IFN-I is higher following infection with African-lineage strains in murine models [40]. While IFN-Is are potent antiviral proteins, they also play important roles in immune activation and for this reason can have dual roles in viral infection outcomes. One hypothesis to explain these data is that decreased virulence, reduced immune activation, and IFN-I sensitivity may be conducive to establishing persistent infections within certain tissues and that rapid, self-limiting virus replication may minimize opportunities to establish infected cell sanctuaries. Indeed, others have suggested that Asian-lineage strains may be better able to establish chronic infection of neural progenitor cells, undergo more efficient vertical transmission, and establish viral reservoirs in the central nervous system, lymph nodes, and gastrointestinal and genitourinary tracts [42,44,45,46,47]. A caveat to all of these studies, including ours, is the divergent passage histories of the strains that are available for study, and we cannot rule out that this drives some of the observed differences. Future studies with larger numbers of low-passage strains involving important target cell types of ZIKV tropism and pathogenesis will be critical in strengthening our understanding of these relationships.

All ZIKV strains were more potently restricted by IFNβ than IFNα at 1000 U/mL. Although IFNα and IFNβ signal through the same heterodimeric IFN receptor (IFNAR), IFNβ has been reported to possess higher binding affinities for IFNAR and can independently bind one IFNAR chain (IFNAR1), which triggers the downstream expression of a unique set of ISGs [48,49]. A combination of these factors likely influences the stronger potency of IFNβ-mediated viral restriction we and others have observed [50]. Of note, we focused on two (IFNα-2a and IFNβ) subtypes that are expressed during ZIKV infection in more relevant cell types, such as human-induced neuroprogenitor cells and the neuroblastoma SH-SY5Y cell line [51], because these IFNs are likely part of the innate antiviral response to ZIKV. However, there are many subtypes of IFN-I that have been shown to have specific and distinct biological effects [49,52,53,54] that may also contribute to IFN-I-induced restriction of ZIKV.

It is noteworthy that while the African-lineage strains were overall less sensitive to IFNα than the Asian-lineage strains, one commonly used African isolate, MR 766, was very sensitive to both IFNα and IFNβ. This may reflect the extensive passage history of MR 766, which could have selected for a virus that is, as a result, not adapted to evade innate immune pressures. Of note, the differences between African-lineage and Asian-lineage groups in terms of both their IFNα and IFNβ sensitivity would have been even stronger if MR 766 were not included in our panel (*p* = 0.0054 for IFNα; *p* = 0.039 for IFNβ). In addition, it is interesting that two of the African-lineage isolates tested, MR 766 and DAK-AR-25, are not very divergent (Figure 1) with only 38 amino-acid differences between the two isolates throughout their entire coding sequences, yet DAK-AR-25 was one of the least IFN-I-sensitive viruses. The key sequence determinants for increased evasion of IFN-I signaling in NS1 and for replication in immunocompetent mice in NS4B [55,56] do not differ between MR766 and DAK-AR-25 and therefore do not explain their differences. Thus, we have identified viruses that show differences in IFN sensitivity that could be leveraged to identify key sequence determinants that predict relative sensitivity to IFN-I.

IFITM3 has recently been reported as an important ZIKV-restricting host factor that blocks an early stage of the ZIKV replication cycle [15,16]. The current findings provide evidence that while IFITM3 has the potential to restrict ZIKV, it does not contribute to the very potent IFN-I-mediated antiviral response to ZIKV in A549 cells. Two ZIKV isolates belonging to each lineage (MR 766 and PRVABC59) that were highly sensitive to IFN-I were not significantly restricted by IFITM3 when it was expressed at physiologically relevant levels. ZIKV replication by IFNβ treatment was potent and unaffected by IFITM3-expression at levels similar to those induced by IFN-I, underscoring the critical contribution of ISGs other than IFITM3 in the IFN-I-mediated restriction of ZIKV. ZIKV restriction also remained unchanged in IFNβ-treated cells in which endogenous IFITM3 had been knocked out, providing further support that endogenous levels of IFITM3 induced by IFN-I do not play a critical role in restricting ZIKV replication. Knocking out IFITM3 expression also abrogated IFITM2 expression. Thus, because there were not significant differences in viral replication between IFITM2/3-deficient cells and control cells, the data is consistent with a prior study reporting that IFITM2 does not play an important role in the IFN-I-induced restriction of ZIKV [15].

We used several experimental approaches to understand why the current findings differ from previous studies that have described an important role for IFITM3 in restricting ZIKV. First, we used two different methods to measure virus, both TCID50 and E-protein staining, as the latter was used in previous studies [15,16]. We did not detect evidence of a role for restriction by IFITM3 at physiological IFN-I-induced levels by either method. Importantly, we used an MOI of 1 and measured viral replication at 48 hpi, which is consistent with most experiments performed in these previous studies. To explore whether IFITM3 is active at earlier stages, we examined virus production at 24 h post-infection and the results were the same as what was observed at 48 h post-infection, suggesting that a transient effect of IFITM3 is not likely to explain differences in results among studies. Rather, our results indicate that the differences between studies are likely due, at least in part, to the use of overexpression of IFITM3 above IFN-I-induced levels, as we show that IFITM3 can inhibit ZIKV when overexpressed even though it does not have an impact at levels similar to those induced by IFN-I.

While our study clarifies that IFITM3 does not contribute significantly to the IFN-I response in A549 cells, we cannot rule out that IFITM3 inhibits ZIKV at baseline levels and/or as a part of the IFN-I response in other cell types, as suggested by results of previous studies showing enhancement of ZIKV infection following shRNA-mediated knock down of IFITM3 (HeLa, HDFa) and IFITM3-knockout (MEF) [15,16]. These studies suggest a possible antiviral role for IFITM3 in inhibiting ZIKV in the absence of IFN and also support that exogenous overexpression of IFITM3 has an antiviral effect in these cell types, as we observed with A549 cells. In these cases, the contribution of IFITM3 was relatively modest (3–10-fold) compared to the several hundred-fold IFN antiviral effect we observed in A549 cells. Future studies that include assessment of the impact of IFITM3 in relation to IFN treatment would help provide a more complete picture of the relative role of this particular ISG in the overall IFN antiviral effect against ZIKV in specific cells. In addition, inactivation studies of the endogenous IFITM3 loci in different cell types with diverse ZIKV strains are needed to better understand whether IFN-induced levels of IFITM3 inhibit specific strains of ZIKV in some cell types.

It is also worth noting that ours is one of only two studies to examine IFITM3-mediated restriction of ZIKV using a complete CRISPR-Cas9-mediated knockout strategy as opposed to a shRNA/siRNA-mediated knockdown approach [15,16]. Interestingly, the CRISPR-Cas9 inactivation study of Spence et al. was conducted in HeLa cells and their combination of knock-out and reconstitution studies show a small (≈7–10-fold) inhibitory effect of IFITM2 and IFITM3 on ZIKV replication in HeLa cells, although it is unclear what fraction of the overall IFN-I response this explained in these cells [17]. Our knock-out studies confirm that in A549 cells, where there is a robust, several order of magnitude inhibitory effect of IFN-I, IFITM3 is not a major player.

Here, we also rule out the possibility that A549s are an anomaly due to unusually low level induction of IFITM3—in fact we found that IFN-I-induced expression of IFITM3 in cells relevant to the neuropathogenesis and vertical transmission of ZIKV, such as Jeg3, SNB-19, and SH-SY5Y cells, are the same or lower than A549 cells. Thus, our findings serve as a caution in interpreting these findings in the context of the overall antiviral effect of IFN-I.

Overall, the results of this study demonstrate that the effects of IFN-I on ZIKV replication in A549 cells are lineage-dependent in our panel of ZIKV strains. The inter-strain variation in IFN-I sensitivity across all viruses in the panel is intriguing and future studies using these strains may identify determinants of IFN-I sensitivity and/or resistance. Finally, the findings suggest that the potent IFN-I effect against ZIKV in A549 cells is not due to IFITM3. Thus, continued investigations of ISGs that restrict ZIKV at physiologically-relevant protein levels, including those that have been recently identified [57,58,59,60], are needed to understand the antiviral ISGs and the innate immune responses important in ZIKV replication and pathogenesis.

## Figures and Tables

**Table 1:** Summary of characteristics of ZIKVs used in this study.

Strain	Lineage	Source	Passage History
MR 766	African	Rhesus (Uganda 1947)	150× mouse brain
			8× Vero cells
IbH 30656		Human (Nigeria 1968)	21× mouse brain
			4× Vero cells
DAK-AR-25		*Aedes africanus* (Senegal 1984)	1× AP61 cells
			1× C6/36 cells
			3× Vero cells
DAK-AR-67		*Aedes taylori* (Senegal 1984)	?× AP61 cells ^1^
			1× C6/36 cells
			2× Vero cells
DAK-AR-71		*Aedes taylori* (Senegal 1984)	?× AP61 cells ^1^
			1× C6/36 cells
			2× Vero cells
PRVABC59	Asian	Human (Puerto Rico 2015)	5× Vero cells
FLR		Human (Columbia 2015)	3× C6/36 cells
H/PAN/2016/BEI-259634		Human (Panama 2016)	4× Vero cells
H/PAN/2015/CDC/259359		Human (Panama 2015)	4× Vero cells

^1^ The number of passages in AP61 cells is unknown.

## Supplementary Materials

The following are available online at https://www.mdpi.com/1999-4915/12/5/503/s1.
