# Supplementary material for: The Robust Restriction of Zika Virus by Type-I Interferon in A549 Cells Varies by Viral Lineage and Is Not Determined by IFITM3
Source: Viruses. 2020 May 2;12(5):503. doi: 10.3390/v12050503 (PMC7290589; doi:10.3390/v12050503)
Supplement: Supplementary file 1 [file viruses-12-00503-s001.pdf]

Table S1. Zika virus sub-amplicon generation primers.

Sub-amplicon 1	
ZikaSeq_7_Fwd	CAAATCACTGTTTGGAGGAATGTCC
ZikaSeq_12_Rev	TTCCAAGGCCCACAGTATGACA
**Sub-amplicon 2**	
ZikaSeq_12_Fwd	GCCTGATATGTGCACTGGC
ZikaSeq_18_Rev	ATCATTAGCAGCGGGACTCCAA

Table S2. Zika virus sub-amplicon sequencing primers.

Name	Sequence (5'-3')
ZikaSeq_9_Fwd	TGACACATGGAGGCTGAAGAGG
ZikaSeq_10_Fwd	TGGAATGGAGATAAGGCCCAG
ZikaSeq_11_Fwd	TTTCAGAGCCAATTGGACACCC
ZikaSeq_12_Fwd	GCCTGATATGTGCACTGGC
ZikaSeq_13_Fwd	GAAGACTGGGAAAAGGAGTGG
ZikaSeq_14_Fwd	TCAAGACAAAGGACGGGGACAT
ZikaSeq_15_Fwd	ATGTGCCATGCCACTTTCACTT
ZikaSeq_9_Rev	CTGCGAAAGTAGCACCCATCA
ZikaSeq_10_Rev	GGGAGAGGAGCATGAACCC
ZikaSeq_11_Rev	CCTTTGAGTATGATCTCTCTCATGGG
ZikaSeq_12_Rev	TTCCAAGGCCCACAGTATGACA
ZikaSeq_13_Rev	TCCTGGTTTTTCCGGCTCC
ZikaSeq_14_Rev	GCTCTCTCTGGGACTTCCACTT
ZikaSeq_15_Rev	CTTCATCAGTCTCTGCACACCC

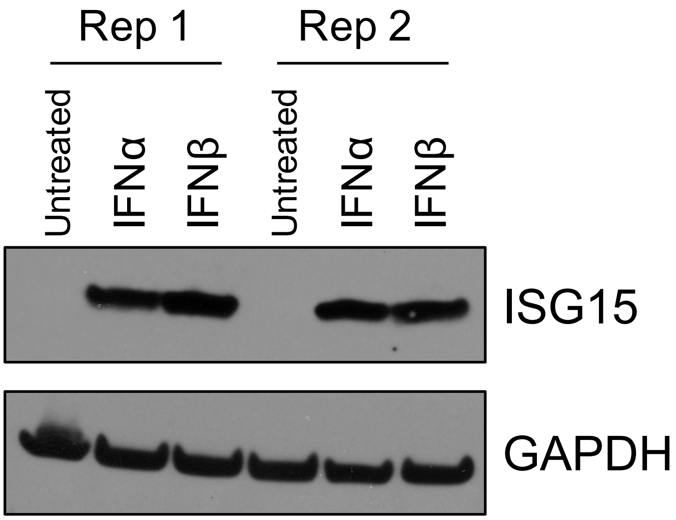

Figure S1. Expression of ISG15 in A549 cells after IFN-I-induction. Western blot analysis of ISG15 expression in A549 cells pretreated with 1000 U/mL IFNα or IFNβ for 24 hours.
